# TGFβ-induced metabolic reprogramming during epithelial-to-mesenchymal transition in cancer

**DOI:** 10.1007/s00018-019-03398-6

**Published:** 2019-12-10

**Authors:** Wan Hua, Peter ten Dijke, Sarantos Kostidis, Martin Giera, Marten Hornsveld

**Affiliations:** 1 Department of Cell and Chemical Biology and Oncode Institute, Leiden University Medical Center, Einthovenweg 20, 2300 RC Leiden, The Netherlands; 2 National and Local Joint Engineering Laboratory for Energy Plant Bio-Oil Production and Application, Key Laboratory of Bio-resources and Eco-environment of Ministry of Education, College of Life Sciences, Sichuan University, Chengdu, People’s Republic of China; 3 Center for Proteomics and Metabolomics, Leiden University Medical Center, Albinusdreef 2, 2300 RC Leiden, The Netherlands

**Keywords:** TGFβ, EMT, Cancer, Signal transduction, Glycolysis, Mitochondrial metabolism, Lipid metabolism, Choline metabolism

## Abstract

Metastasis is the most frequent cause of death in cancer patients. Epithelial-to-mesenchymal transition (EMT) is the process in which cells lose epithelial integrity and become motile, a critical step for cancer cell invasion, drug resistance and immune evasion. The transforming growth factor-β (TGFβ) signaling pathway is a major driver of EMT. Increasing evidence demonstrates that metabolic reprogramming is a hallmark of cancer and extensive metabolic changes are observed during EMT. The aim of this review is to summarize and interconnect recent findings that illustrate how changes in glycolysis, mitochondrial, lipid and choline metabolism coincide and functionally contribute to TGFβ-induced EMT. We describe TGFβ signaling is involved in stimulating both glycolysis and mitochondrial respiration. Interestingly, the subsequent metabolic consequences for the redox state and lipid metabolism in cancer cells are found to be in favor of EMT as well. Combined we illustrate that a better understanding of the mechanistic links between TGFβ signaling, cancer metabolism and EMT holds promising strategies for cancer therapy, some of which are already actively being explored in the clinic.

## Background

Increasing evidence shows that the metabolic dysregulation of cancer cells is related to the epithelial-to-mesenchymal transition (EMT) program [1, 2]. In this review, we address the current understanding of metabolic alterations during transforming growth factor-β (TGFβ)-induced EMT and cancer metastasis. In particular, we focus on documented changes in glycolysis, the metabolism of lipids, mitochondrial and choline-associated pathways, and their functional roles.

**Fig. 1:**
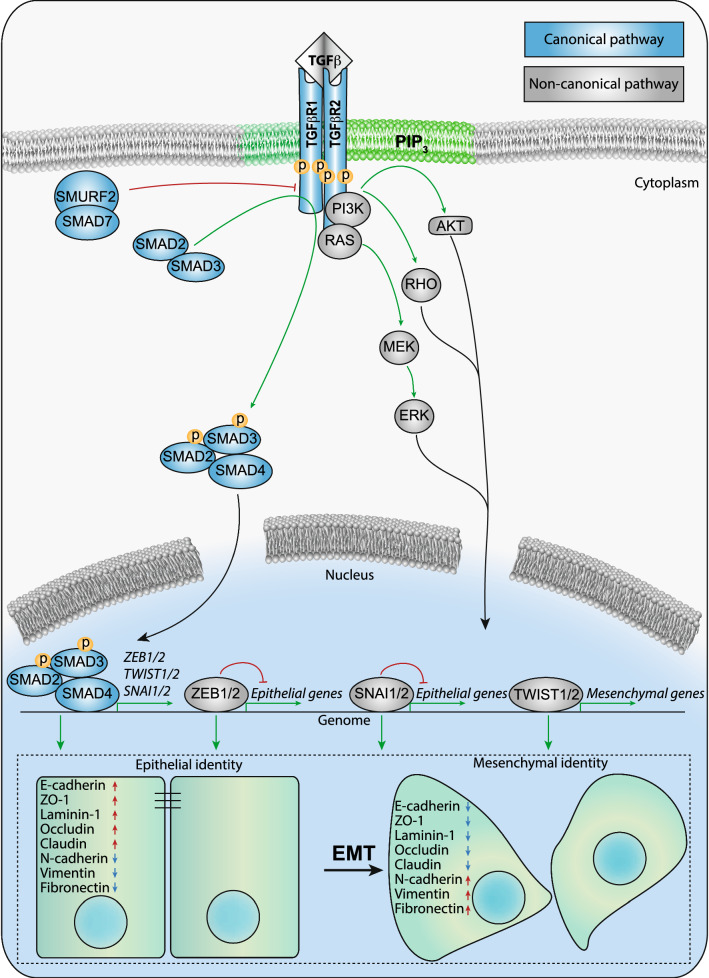
TGFβ-induced epithelial-to-mesenchymal transition. TGFβ signaling can be activated by TGFβ ligands and activate both canonical (blue) and non-canonical pathways (grey). In the canonical SMAD pathway, TGFβR2 activated TGFβR1 phosphorylates SMAD2 and SMAD3, which subsequently form a complex with SMAD4 and translocate to the nucleus. Upon DNA binding, the SMAD complex activates drivers of EMT (SNAI1/2, ZEB1/2 and TWIST1/2) as well as negative feedback component SMAD7. SMAD7 negative feedback regulates TGFβ signaling via recruiting the E3 ubiquitin ligase SMURF2 to activated TβR1/2 receptors and then targets the receptors for degradation. SMAD-independent activation of PI3K/AKT, RHO and RAS/MAPK pathways by TGFβ are non-canonical effectors also involved in EMT and survival. Cancer cells undergoing EMT lose epithelial properties, including cell–cell adhesion, apical–basal polarity and the decrease expression of epithelial genes, i.e. E-cadherin, ZO-1, laminin-1, occludin, claudin. Subsequently, cancer cells acquire migratory mesenchymal characteristics, involving tight-junction dissociation and increased expression of mesenchymal markers, i.e. N-cadherin, vimentin and fibronectin

The TGFβ family is a family of cytokines that act on many different cell types in a pleiotropic manner [3, 4]. TGFβ is secreted by many cell types, and high levels are present in platelets and bone. Three TGFβ isoforms, i.e., TGFβ1, TGFβ2 and TGFβ3, are present in mammals. Differences in their biological functions are predominantly attributed to different spatiotemporal expression patterns [5], and exogenous ligand addition to the three TGFβ isoforms has redundant cellular effects. Therefore, in this review, we will refer to TGFβ and not mention a specific isoform unless required. TGFβ signaling is involved in embryonic development and adult tissue homeostasis through the regulation of cell proliferation, survival and differentiation [6, 7]. TGFβ elicits its cellular effects via single transmembrane-spanning TGFβ type I and type II receptors (TGFβR1 and TGFβR2) [8, 9]. Both receptors are endowed with intrinsic serine/threonine kinase activity. TGFβ induces the formation of heteromeric complexes in which the TGFβR2 kinase phosphorylates TGFβR1 [10]. The activated TGFβR1 transduces the signal into the cell by phosphorylating receptor-regulated (R-)SMADs, SMAD2 and SMAD3. These R-SMADs are canonical intracellular effectors of TGFβ. Activated SMAD2 and SMAD3 partner with the SMAD4 transcription factor, and upon their nuclear translocation, heteromeric SMAD complexes orchestrate the regulation of a plethora of targets (Fig. 1). SMAD3 and SMAD4, but not SMAD2, bind directly to DNA [11]. The multifunctionality of TGFβ/SMAD signaling events is mediated in part by cell type-dependent interactions with other DNA-binding transcription factors, co-activators and repressors [3, 12]. Canonical target genes include the extracellular matrix protein *plasminogen activator inhibitor*-*1* (*PAI*-*1*) and inhibitory *SMAD7*. SMAD7 regulates the intensity and duration of TGFβ signaling through negative feedback. SMAD7 recruits the E3 ubiquitin ligase SMURF2 to activated TGFβR1/2 receptors and subsequently targets the receptors for degradation [13, 14]. In addition to the canonical SMAD pathway, TGFβ can also activate non-SMAD signaling pathways, such as the Rho GTPase family, the phosphatidyl inositol 3 kinase (PI3K/AKT), and mitogen-activated protein kinase (MAPK) signaling pathways [15–17] (Fig. 1).

TGFβ signaling plays a dual role in cancer progression [18, 19]. In the early stages of cancer, TGFβ acts as a tumor suppressor by inhibiting proliferation and the induction of apoptosis in healthy and premalignant cells [20–22]. Stimulation of TGFβ signaling represses the cell cycle in G1 phase by the inhibition of cyclin-dependent kinases (CDKs). TGFβ also downregulates c-MYC expression, a pro-oncogenic transcription factor that activates the expression of many pro-proliferative genes [23, 24]. In later stages of tumorigenesis, TGFβ-induced cytostatic responses are mitigated by activation of oncogenes or loss of tumor suppressor genes. TGFβ, frequently secreted in high amounts by cancer and surrounding cells of the tumor microenvironment, then switches its action into a tumor promoter not only by stimulating angiogenesis and immune evasion but also by stimulating a process called epithelial–mesenchymal transition (EMT) in cancer cells [25–27]. Also, increased expression of factors such as 14-3-3ζ and PSPC1 mediate the switch of TGFβ signaling from tumor suppressing to promoting cancer progression [28, 29].

## TGFβ-induced epithelial–mesenchymal transition

EMT is characterized by morphological changes in which apical–basal polarized epithelial cells are converted into cells with a mesenchymal spindle-shaped phenotype [30]. EMT is a process of key importance for embryogenesis, and organ development. EMT has been causally linked to cancer invasion and metastasis [31–33] as well as chemotherapy resistance [34] and TGFβ is a pivotal driver of these multistep processes.

Numerous studies have shown that the activation of EMT is fundamental to malignant progression and metastatic dissemination [32, 35]. EMT is defined by the loss of cell–cell adhesion accompanied by the decreased expression of E-cadherin, cytokeratin, ZO-1, laminin-1, occludin and claudin, and the acquisition of a motile and migratory phenotype with the increased expression of N-cadherin, vimentin, and fibronectin, ultimately facilitating the migration of cancer cells [31, 36]. EMT is a dynamic and multistep process closely linked to the interaction between multiple cytokine and inflammatory factors, transcription factors and cellular pathways. Expression of genes that inhibit an epithelial cell phenotype and activate a mesenchymal phenotype involves the transcription factors SNAI1/2, ZEB1/2 and TWIST1/2. In the early stages of EMT, these transcription factors are activated, bind to the E-box in the E-cadherin promoter region and downregulate the expression level of E-cadherin, subsequently reducing epithelial identity [37] (Fig. 1). Increasing evidence demonstrated that EMT is not a binary process, but that partial states exist in which epithelial and mesenchymal markers are co-expressed by cancer cells. These hybrid states are associated with an aggressive phenotype as they confer high plasticity and stemness properties to cancer cells [38–40]. While in most cancer cells, TGFβ-induced EMT is associated with pro-oncogenic response, in pancreatic cancer cells with an intact TGFβ/SMAD4 pathway, it was found associated with apoptosis in a KLF5 repression-dependent manner. In the absence of SMAD4, KLF5 cooperates with SOX4 to promote tumor progression in pancreatic cancer cells [41].

The canonical TGFβ/SMAD pathway plays a pivotal role in mediating EMT. TGFβ-induced SMADs bind to the promotors of *SNAI1*, *TWIST1/2* and *ZEB1* and increase their expression [42, 43]. In addition, SMADs can interact and cooperate with SNAI1/2 in a common transcriptional repressive complex that promotes EMT [44]. Epigenetic changes induced by TGFβ/SMAD signaling also contribute to EMT [45, 46]. The non-SMAD signaling pathways of TGFβ can also facilitate epithelial plasticity, sometimes in collaboration with the SMAD pathway [47] (Fig. 1). For example, activation of the PI3K/AKT pathway was required for TGFβ-induced EMT, inhibition of mTOR, a downstream protein kinase of PI3K/AKT signaling, reduced cell migration, adhesion, and invasion that accompany TGFβ-induced EMT of namru murine mammary gland (NMuMG) cells [48, 49]. Moreover, AKT-induced TWIST phosphorylation promoted TGFβ2 transcription and TGFβ receptor activation, and stimulates EMT [50].

It is worth noting that TGFβ-induced EMT can also be a reversible process in cell culture. Upon TGFβ removal, mesenchymal cells can revert back to an epithelial phenotype. Recent findings indicated that a chronic TGFβ treatment induced a stable mesenchymal state in mammary epithelial and breast cancer cells that is different to the reversible EMT upon short-term TGFβ exposure. This stable EMT phenotype was associated with an increased tumor stemness and cancer drug resistance that is susceptible to mTOR inhibition [51].

## Metabolic reprogramming in tumorigenesis and EMT

Metabolic reprogramming is a hallmark of cancer that contributes to tumorigenesis and disease progression [52]. Cancer cells rewire metabolic pathways to satisfy their requirement for ATP production, biomass generation and redox balance. The Warburg effect is the most recognized metabolic phenotype observed in cancers. Cancer cells upregulate the uptake of glucose and shift their metabolism from oxidative phosphorylation towards glycolysis, even under aerobic conditions [53, 54]. Although ATP production from glycolysis is very inefficient (2 mol ATP per mol glucose compared to 36 mol ATP per mol glucose in glycolysis and oxidative phosphorylation, respectively), tumors experience advantages in their growth and development from high levels of glycolysis for several reasons. First, high glycolytic rates can increase the tolerance of cancer cells to oxygen fluctuations. Second, as lactate, the final product in glycolysis, can contribute to tumor acidity, the accumulation of lactate promotes immune escape and tumor invasion [55, 56]. Third and most importantly, aerobic glycolysis satisfies the demand of rapidly proliferating cancer cells for macromolecular anabolism as large amounts of intermediate metabolites from glycolysis are shunted into different biosynthetic pathways [53, 57, 58].

A recent study found that the Warburg effect contributed to cancer anoikis resistance, which is a prerequisite for tumor metastasis. The shift of ATP generation from oxidative phosphorylation to that from glycolysis protects cancer cells against reactive oxygen species (ROS)-mediated anoikis [59, 60]. As mentioned above, the aberrant activity of oncogenes and tumor suppressors, such as hypoxia-inducible factor 1 (HIF-1), AKT, MYC, p53 and phosphatase and tensin homolog (PTEN), directly affect metabolic pathways, particularly glycolysis [58, 61, 62]. In addition, enhanced glycolysis accompanied by increased lactate fermentation and alleviated mitochondrial respiration protects cancer cells against oxidative stress, favoring tumor metastasis.

The molecular mechanisms of metabolic reprogramming in cancer cells are complex. Metabolic alterations in cancer have been found to be related to the mutation or abnormal expression of oncogenes or tumor suppressors. For instance, KRAS mutations can alter the metabolic flux of pancreatic cancer cells, selectively decompose glucose through the non-redox pentose phosphate pathway, and promote pentose production and nucleic acid synthesis [63]. Aberrant expression of metabolic enzymes is also a key factor for metabolic reprogramming in cancer that is often regulated by certain oncogenes or tumor suppressor genes [64]. For example, PI3K, KRAS and hypoxia-inducible factor (HIF) are responsible for the upregulation of glucose transporter 1 (GLUT1) [65–67]. While it remains to be experimentally tested, it is interesting to take into account that PI3K/AKT and KRAS/MEK/ERK pathways can also be triggered as part of non-canonical TGFβ-signaling and, therefore, might contribute to TGFβ-associated metabolic effects (Fig. 1).

Moreover, metabolic enzyme mutation and dysregulated metabolic enzyme activity can affect cellular metabolism [68]. As cancer cells rely on altered metabolism to support cell proliferation and survival, metabolic pathways are potential therapeutic targets. Recent findings indicate that metabolic changes and EMT are intertwined. While metabolic alterations possibly induce EMT, EMT may also lead to metabolic changes [1, 2]. Notably, a group of 44 metabolic genes named the “mesenchymal metabolic signature” (MMS) genes were found to exhibit increased expression levels in high-grade carcinoma cell lines. Expression of these MMS genes is correlated tightly with mesenchymal markers and was shown to be upregulated upon the activation of EMT by TWIST1 expression in human mammary epithelial cells. Among these genes, dihydropyridine dehydrogenase (DPYD), an enzyme involved in pyrimidine degradation, was found to be essential for EMT. The role of TGFβ in regulating DPYD remains to be investigated. Importantly, this finding implicates that EMT and metabolic reprogramming are intertwined [69]. Increasing evidence indicates that metabolic alterations influence the EMT process through affecting epigenetic modifications [62]. In this context, the intermediary metabolites of glycolysis and oxidative phosphorylation, such as acetyl-CoA, nicotinamide adenine dinucleotide; NAD^+^/NADH and flavin adenine dinucleotide (FAD), are used as cofactors to promote epigenetic modifications [62, 70]. While it is clear that EMT and cellular metabolism are interlinked, the knowledge on the role of TGFβ signaling, as well as alternative pathways that can induce EMT, in metabolic reprogramming during EMT is still limited and scattered over various cancer models. In the following sections, we describe the emerging relationships between TGFβ-induced EMT and reprogramming of glycolysis, mitochondrial respiration, lipid and choline metabolism.

## TGFβ signaling stimulates glycolysis

Aerobic glycolysis enhances the proliferation and survival of tumor cells and provides the basis for tumor cell metastasis and invasion by supplying a large number of metabolic precursors. Simultaneously, the shift from oxidative phosphorylation to glycolysis reduces mitochondrial metabolism, accompanied by decreased ROS production, and was shown to result in anoikis resistance and tumor metastasis [59, 60]. Moreover, the preferential use of aerobic glycolysis leads to the accumulation of lactate in the tumor microenvironment, facilitating the stabilization of HIF-1 and subsequently upregulating the expression of vascular endothelial growth factor (VEGF), resulting in angiogenesis and the stimulation of cell migration [71]. A recent study suggested that extracellular lactate induces SNAI1 and EMT through directly remodeling the extracellular matrix and releasing activated TGFβ1. Furthermore, high extracellular lactate levels can contribute to immune evasion, thereby promoting tumor growth and metastasis [72].

**Fig. 2:**
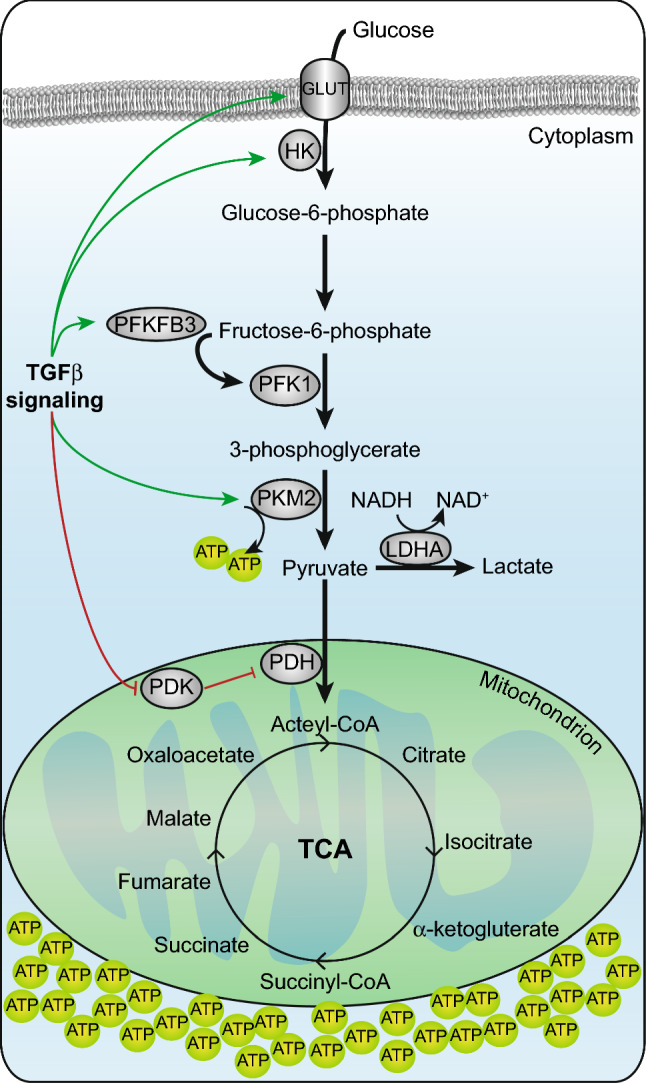
Glycolysis is enhanced during TGFβ-induced EMT. Increased activity of glycolytic enzymes, GLUT1, hexokinase 2 (HK2), 6-phosphofructo-2-kinase/fructose-2,6-biphosphatase 3 (PFKFB3), 6-phosphofructo-1-kinase (PFK1), pyruvate kinase M2 (PKM2), lactate dehydrogenase type A (LDHA) result in a high glycolytic rate during TGFβ-triggered EMT in cancer cells. While glycolysis is stimulated and lactate is produced, pyruvate dehydrogenase kinase 4 (PDK4), a negative regulator of pyruvate dehydrogenase (PDH) is inhibited by TGFβ signaling to also allow pyruvate into the TCA cycle

Glycolysis has been found to be upregulated in malignant cancer cells. For instance, highly metastatic MDA-MB-231 breast cancer cells have a much higher glycolytic efficiency than nonmetastatic MCF-7 breast cancer cells [55]. Enhanced glycolysis is mainly due to the increased expression or activity of key enzymes. In recent years, tumor targeting by inhibiting the activity of key enzymes in the glycolytic pathway has been explored to decrease proliferation, invasion and metastasis. The levels of glucose transporter 1 (GLUT1) have been demonstrated to increase upon TGFβ stimulation in mesangial cells, breast cancer cells, glioma cells and pancreatic ductal adenocarcinoma [73–76] (Fig. 2). GLUT1 expression was correlated with EMT markers, including E-cadherin and vimentin, and accompanied by increased glucose uptake during TGFβ-induced EMT in breast cancer cells [74]. Furthermore, glucose transporter 3 (GLUT3) also showed higher expression in mesenchymal cells compared to that in epithelial cells among non-small-cell lung carcinoma (NSCLC) cell lines and was also upregulated during TGFβ-induced EMT in H2122 lung cancer cells (Fig. 2).

GLUT3 was identified as a transcriptional target of ZEB1 that facilitates increased cancer cell motility and invasion [77]. TGFβ1 was also shown to upregulate the expression of the gene-encoding hexokinase 2 (HK2), which phosphorylates glucose to produce glucose-6-phosphate (G6P) in the first step in the glycolytic pathway [73] (Fig. 2). HK2 was recently reported to promote pancreatic ductal adenocarcinoma (PDAC) metastasis by mediating lactate production. Moreover, HK2 was also shown to play a key role in the metastatic progression of neuroblastoma [78, 79]. Another enzyme, 6-phosphofructo-2-kinase/fructose-2,6-biphosphatase 3 (PFKFB3), is involved in glycolytic alteration upon TGFβ-induced EMT. One of the rate-limiting steps in glycolysis is the 6-phosphofructo-1-kinase (PFK1) catalyzed conversion of fructose-6-phosphate (F6P) to fructose-1,6-bisphosphate (F1,6P2). PFKFB3 is responsible for the synthesis of fructose-2,6-bisphosphate (F2,6P2), a potent allosteric activator of PFK1, which stimulates glycolysis [80, 81]. TGFβ1 enhanced PFKFB3 expression and stimulated glycolysis in Panc1 pancreatic cells. *PFKFB3* silencing inhibited TGFβ-induced invasion in this cell line by repressing the expression of *SNAI1* [82]. In addition, PFKFB3 was also upregulated by TGFβ1 in glioma cells, resulting in an increase in fructose 2,6-bisphosphate, glucose uptake, glycolytic flux and lactate production [73] (Fig. 2). Pyruvate kinase M2 (PKM2) is overexpressed in more than 70% of human cancers [83]. This enzyme catalyzes the last step of glycolysis, in which phosphoenolpyruvate (PEP) is dephosphorylated to pyruvate with ATP production. Upon TGFβ-induced EMT of A549 lung adenocarcinoma cells, the expression of PKM2 increases. When levels of PKM2 were decreased by aspirin-triggered resolvin D1 (AT-RvD1), which is well known to be associated with inflammation, TGFβ1-induced EMT was mitigated [84]. Moreover, in colon cancer cells, PKM2 interacts with TGFβ-induced factor homeobox 2 (TGIF2) during TGFβ-induced EMT (Fig. 2). TGFIF is a transcriptional repressor of TGFβ signaling, and the complex between PKM2 and TGIF2 promotes histone H3K9 deacetylation, resulting in a decrease in E-cadherin transcription [85].

The Warburg effect is characterized by increased glucose uptake and glycolytic activity and the accumulation of lactate in cancer [53]. Interestingly, lactate levels are directly related to cancer metastasis [72, 86]. Cancer cells can metabolize lactate as an energy source and also obtain lactate from cells in the tumor microenvironment, thereby maintaining their acidic environment, leading to apoptosis resistance and increased survival and proliferation [87, 88]. The increased production of lactate occurs during TGFβ-induced EMT [73, 76, 82]. Lactate dehydrogenase type A (LDHA) plays an important role in catalyzing the conversion of pyruvate into lactate. Lactate was shown to promote glioma migration by the TGFβ2-dependent regulation of matrix metalloproteinase-2 (MMP2) [89].

At the end of the glycolytic pathway, pyruvate dehydrogenase kinase (PDK) is an enzyme that inactivates pyruvate dehydrogenase (PDH), which is responsible for converting pyruvate to acetyl-CoA in the mitochondria. Acetyl-CoA then enters the citric acid cycle (TCA) to generate ATP and electron donors, including NADH. In lung cancer cells, downregulation of PDK4 resulted in a shift from glycolysis to oxidative phosphorylation (OXPHOS). TGFβ represses PDK4 expression, and PDK4 inhibition was sufficient to drive EMT and promote erlotinib resistance in epidermal growth factor receptor (EGFR) mutant lung cancer cells (Fig. 2). Consistent with these results, the overexpression of PDK4 partially blocked TGFβ-induced EMT [90].

Collectively, these results show that TGFβ stimulates glycolysis while inducing EMT transcriptional programs in a synergistic manner. The transition from oxidative metabolism to glycolysis may benefit tumor metastasis due to the accumulation of cellular building blocks, the rapid generation of ATP and resistance to oxygen fluctuations. The observation that PDK4 is antagonized by TGFβ signaling, however, implicates that flexibility in metabolic routing is a prerequisite for EMT as pyruvate is still allowed, and essential, to shunt into the TCA cycle for EMT to complete.

## The TCA cycle is active during EMT

Mitochondria not only are the main energizing organelles in eukaryotes but also play a crucial role in anabolic metabolism by providing intermediates to produce glucose, amino acids, lipids and nucleotides. Therefore, mitochondrial dysfunction is closely related to many diseases, including cancer. Accumulating evidence indicates that disordered mitochondrial metabolism is associated with tumor invasion and metastasis [91–93]. The TCA cycle, one of the main pathways within mitochondria, plays a key role in cell proliferation and provides important intermediates for amino acid, nucleic acid and lipid biosynthesis. Some enzymes involved in the TCA cycle are mutated or dysfunctional in different cancers, i.e. fumarate hydratase (FH), succinate dehydrogenase (SDH), and isocitrate dehydrogenase (IDH). Although these mutations have been directly linked to EMT, the role of TGFβ in regulating TCA intermediates during EMT induction remains to be fully explored [2, 94–99].

**Fig. 3:**
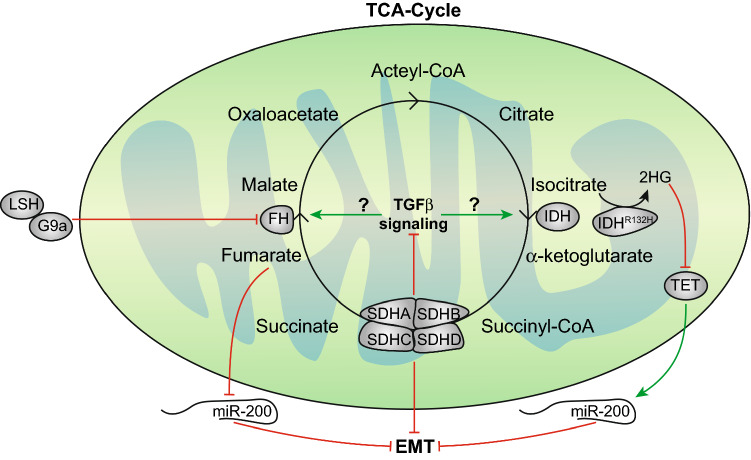
The TCA cycle is active in TGFβ-induced EMT. The TCA cycle occurs in the matrix of mitochondria and is a key metabolic pathway for providing intermediates to generate glucose, amino acids, lipids, nucleotides and most importantly, ATP. Altered activity of fumarate hydratase (FH), succinate dehydrogenase (SDH), and isocitrate dehydrogenase (IDH) are connected to EMT. The chromatin-remodeling factor lymphoid-specific helicase (LSH) can promote EMT through recruiting the epigenetic silencer factor G9a to suppress FH expression. Furthermore, the accumulation of fumarate also enhances EMT by inhibiting miR-200. Reduced levels of SDHB stimulate EMT by activating the TGFβ signaling pathway. IDH converts isocitrate to α-ketoglutarate but the oncogenic IDH^R132H^ leads to the production of the oncometabolite 2HG. 2HG interferes with chromatin remodeling enzymes and downregulates miR-200 expression via inhibition of TET, subsequently stimulating EMT

FH is a metabolic enzyme responsible for converting fumarate to malate. FH mutations have been discovered in hereditary leiomyomatosis, renal cell cancer, pheochromocytoma and paragangliomas [97, 100]. A recent study demonstrated the relationship between FH and EMT in nasopharyngeal carcinoma (NPC). The chromatin-remodeling factor lymphoid-specific helicase (LSH), the expression of which is controlled by the Epstein–Barr virus-encoded protein LMP1, was increased in NPC. LSH can promote EMT via recruiting the epigenetic silencer factor G9a to repress FH transcription. FH dysfunction resulted in decreased malate and increased α-ketoglutarate (α-KG) levels. Increased α-KG levels altered nuclear factor κ-B kinase α (IKKα)-dependent EMT gene expression by inducing the binding of IKKα to the promoter region of EMT-related genes. This leads to migration and invasion in vitro and tumor growth and metastasis in vivo [101]. Furthermore, fumarate accumulation in FH-deficient renal cancer cells elicited EMT by inhibiting the ten–eleven translocation (TET)-dependent demethylation of the anti-metastatic mRNA miR-200, which suppressed the expression of the EMT transcription factors SNAI2, ZEB1 and ZEB2 [96, 102, 103] (Fig. 3).

Another TCA cycle enzyme linked with EMT is SDH, which is involved in the conversion of succinate to fumarate in the respiratory chain. The SDH complex is composed of four subunits: SDHA (a flavoprotein), SDHB (an iron–sulfur protein), and the two membrane anchor subunits SDHC and SDHD. Mutations in gene-encoding subunits of this enzyme complex have been reported in a subset of human cancers [104, 105]. Decreased SDHB expression has been reported in both human colorectal cancer (CRC) samples and CRC cell lines, and reduced SDHB activity has been correlated with a more advanced clinical lymphatic and distant metastatic phenotype. Further research found that the knockdown of SDHB contributed to cell invasion and migration via EMT by activating the TGFβ signaling pathway through upregulation of the tight junction transcriptional repression complex SNAI1-SMAD3/SMAD4 [106]. The knockdown of SDHB resulted in a hypermethylated epigenome, which was sufficient to trigger EMT in mouse ovarian cancer cells. In addition, SDHB downregulation resulted in succinate accumulation, altered center carbon metabolism and induced mitochondrial dysfunction [94] (Fig. 3).

IDH has also been associated with cancer development and EMT. IDH catalyzes the oxidative decarboxylation of isocitrate into α-ketoglutarate (α-KG). Furthermore, IDH exists as three isoforms: cytosolic IDH1 and mitochondrial IDH2, which are both NADP^+^ -dependent enzymes, and mitochondrial IDH3, which is an NAD^+^ -dependent  enzyme. Mutations in *IDH* are associated with the development of multiple cancers [107–109]. Mutations in *IDH1* and *IDH2* lead to the production of *R*-2-hydroxyglutarate (2HG) from isocitrate instead of α-KG. 2HG and α-KG are highly similar in structure [110], and 2HG is a competitive inhibitor of α-KG-dependent dioxygenases which mainly induces the histone demethylase and DNA demethylase 10–11 TET proteins. The cellular accumulation of 2HG is closely related to the occurrence and development of cancer [111]. The production of 2HG inhibits the activity of α-KG-dependent dioxygenase, leading to impaired histone modification and DNA demethylation [112]. 2HG promotes EMT by increasing histone H3 methylation of the *ZEB1* promoter region to upregulate the expression of *ZEB1* and downregulate *miR*-*200* expression [95, 113]. Together, ZEB1, miR-200 and TGFβ are part of an autocrine signaling network that stimulates EMT [114]. It might, therefore, be expected that TGFβ signaling synergizes with IDH mutations in the stimulation of invasive cancers (Fig. 3).

Taken together, there is sufficient evidence that TCA cycle metabolites are involved in EMT, but determining the causal relation between metabolite accumulation in one step of the TCA cycle to EMT is challenging. For example, accumulation of fumarate in FH mutant cells is also expected to increase the levels of its precursors such as succinate and α-ketoglutarate. Changes in TCA metabolites can have broad epigenetic effects; therefore, it becomes hard to exactly pin point the exact event that triggers EMT and elucidate the mechanism behind this. It is clear, however, that elucidation of TCA metabolite effects in TGFβ-induced EMT holds promising insights into precision-targeting metastatic cancers in the future. Interestingly, TGFβ-induced EMT seems to trigger both glycolysis and mitochondrial activity. This is expected to be a requirement to provide the cell with sufficient ATP to become motile. Indeed, by performing single-cell ATP assays, it was shown that TGFβ-induced EMT results in a spike in ATP levels [115]. Putting ATP production in overdrive comes with a cost, however, as it coincides with increased production of ROS and reduced lipogenesis for which TCA intermediates are required.

## Redox regulation of TGFβ-mediated EMT

ROS are highly reactive molecules and the most common forms of cellular ROS are hydrogen peroxide (H_2_O_2_), superoxide (O_2_^•−^) and hydroxyl radicals (HO^•^). Especially, H_2_O_2_ can function as a second messenger by oxidizing target proteins, hereby altering their function or binding partners. The main source of ROS is the ATP-producing electron transport chain in mitochondria. ROS play an important role in cell signaling and homeostasis [116]. ROS levels in tumor tissues are higher compared to normal tissue and ROS affect many aspects of tumorigenesis [117]. It has been suggested that ROS production is induced by TGFβ to mediate cell proliferation, apoptosis and EMT in cancer [118].

**Fig. 4:**
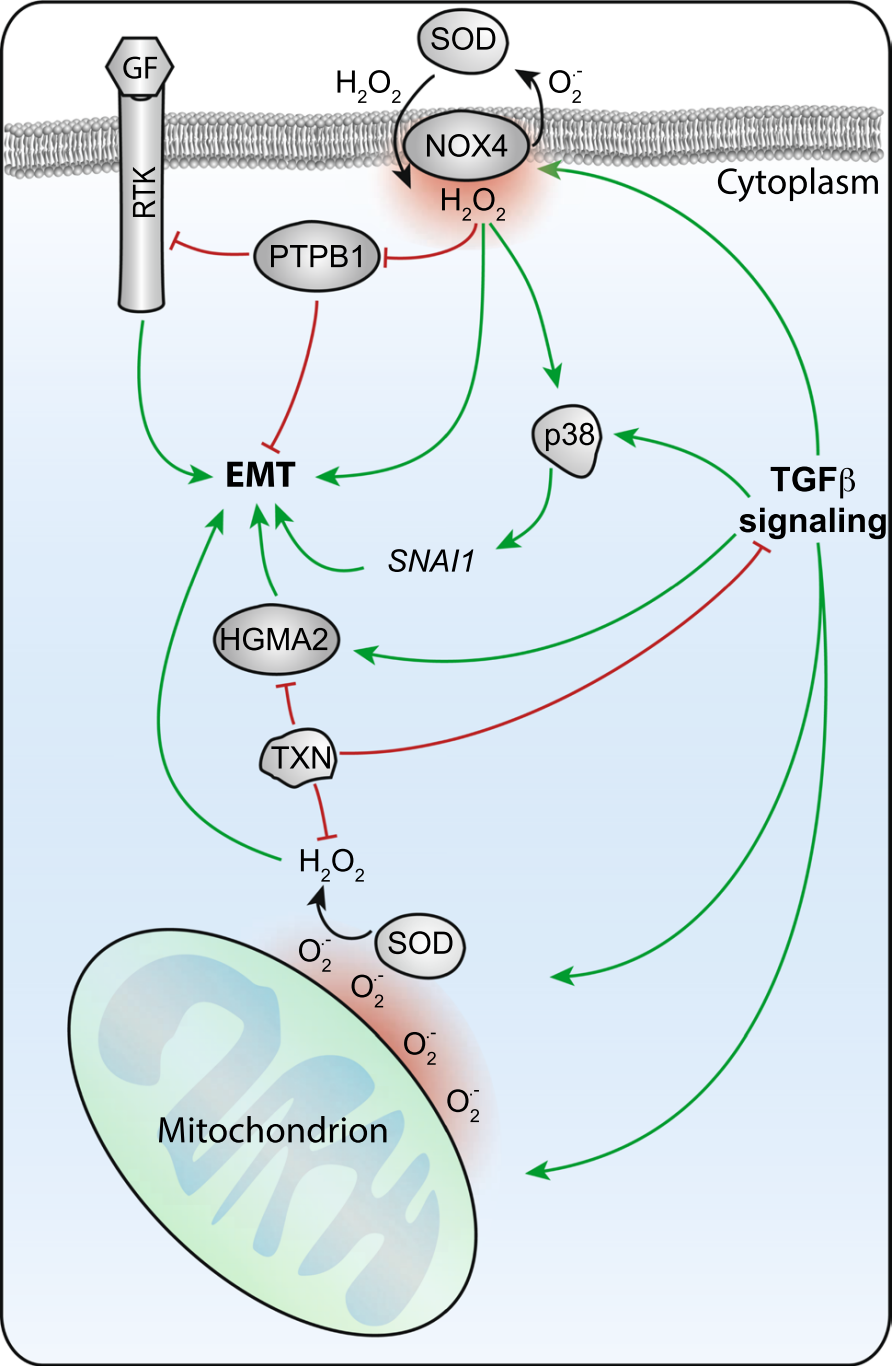
Redox regulation of TGFβ-mediated EMT. TGFβ increases O_2_^•−^ production by mitochondria, which stimulates EMT. H_2_O_2_ dismutation from O_2_^•−^ released from mitochondrial by superoxide dismutase 2 (SOD2) is counter balanced by thioredoxin (TXN) pathways. TXN interferes with TGFβ-induced EMT through reduction of H_2_O_2_ and suppression of high-mobility group AT-hook 2 (HMGA2) transcription factor expression. In addition, TGFβ also induces NADPH oxidase 4 (NOX4) to produce O_2_^•−^ followed by dismutation into H_2_O_2_. H_2_O_2_ derived from NOX4 inhibits protein–tyrosine phosphatase 1B (PTP1B), a negative regulator of EMT and mitogenic receptor tyrosine kinase (RTK) signaling. Additionally, NOX4 derived H_2_O_2_ stimulates TGFβ-induced p38MAPK activation, which enhances EMT by promoting SNAI1 expression

The primary source from which ROS production is stimulated during TGFβ-induced EMT is the mitochondria and found essential for this process [119, 120]. Conversely, it has been demonstrated that mitochondrial thioredoxin (TXN2) repressed TGFβ-induced EMT via different mechanisms. First, TXN interferes with ROS directly as these are important players in the antioxidant pathways that reduce free radicals. Second, TXN antioxidant responses counteract expression of high-mobility group AT-hook 2 (HMGA2), which is a positive regulator of EMT [119, 120]. Furthermore, the mitochondrial enzyme superoxide dismutase 2 (SOD2), which catalyzes O_2_^•−^ radicals to H_2_O_2_ and oxygen, has been found upregulated and required in TGFβ-induced EMT in human oral and esophageal epithelial cell lines [121]. This implicates that the generation of O_2_^•−^ and subsequent H_2_O_2_ by mitochondria is indeed actively stimulated by TGFβ during EMT, but kept within a concentration range that is not harmful for the cell (Fig. 4).

An additional source of TGFβ-induced ROS is NADPH oxidase 4 (NOX4), which belongs to the family of NADPH oxidase enzymes. NOX4 is localized at the cell membrane and NOX4-produced ROS is considered an important redox signal transducer in TGFβ-induced EMT in pancreatic cancer and glioblastoma cells [122–124]. ROS derived from NOX4 oxidize and thereby inhibit protein-tyrosine phosphatase 1B (PTP1B), a known negative regulator of mitogenic signaling and cell migration. Prevention of NOX4-generated ROS by chemical inhibition repressed TGFβ-induced EMT [123]. Additionally, NOX4 stimulates TGFβ-induced p38MAPK activation, which enhances EMT by promoting *SNAI1* expression [123]. Others have shown that NOX4 is increased during TGFβ-induced EMT in a SMAD3-dependent manner in breast cancer cells [125] (Fig. 4).

Combined, the generation of ROS from mitochondria and NOX4 in response to TGFβ-induced EMT increases the cellular redox state towards a more oxidative milieu. The consequential redox signaling effects on molecular signal transduction subsequently give rise to an EMT permissive state. Increased redox signaling in renal tubular epithelial cells stimulates phosphorylation of SMAD2 and MAPK, and the expression of mesenchymal markers, which could be counteracted by addition of antioxidants [120]. Similarly, extracellular signal-regulated kinase (ERK) inhibition could repress TGFβ and H_2_O_2_-induced EMT, further supporting the important role of ROS in TGFβ-induced EMT via the receptor tyrosine kinase pathways [120]. In accordance, another recent finding indicated H_2_O_2_-enhanced TGFβ-mediated EMT via SMAD and MAPK kinase (MEK)/ERK signaling in MCF-10A human mammary epithelial cells [126] (Fig. 4).

Nuclear factor E2-related factor 2 (NRF2) is a critical transcriptional factor that plays a key role in cellular responses to high levels of ROS via stimulating many antioxidant proteins including glutathione S-transferase (GST), NAD(P)H, and quinone oxidoreductase 1 (NQO1) [127]. NRF2 activation contributed to EMT by mediating a decrease in E-cadherin expression and stimulates TGF-β1-induced SMAD2/3 activity in a pancreatic ductal adenocarcinoma cell line [128]. In addition, NRF2 knockdown promoted TGF-β1-induced EMT via upregulating SNAIL expression in pulmonary fibrosis [129].

Due to the complex nature of redox signaling on the level of signal transduction and limited experimental tools to study it, the mechanism on how these signals become specific and mechanistically influence signaling proteins is still in the process to be fully elucidated. It is clear, however, that redox signaling and changing the cellular redox state is not only simply a negative consequence of increased ATP production, but also an important aspect of TGFβ-induced EMT. Altering the redox balance is a cancer-targeting strategy that is currently intensively studied and understanding redox regulation of TGFβ-induced EMT might unveil promising targets to interfere metastasis formation.

## Lipid metabolism in TGFβ-induced EMT

Lipids have traditionally been defined as molecules that are insoluble in water and soluble in organic solvents. As this property can be found in a number of biological molecules, a classification system based on chemical and biochemical principles has been established [130]. According to their function, lipids can roughly be divided into storage lipids, such as triglycerides and cholesterol esters, and signaling lipids, such as eicosanoids or steroid hormones. Among the metabolic pathways that are reprogrammed in cancer, lipid metabolism has received less attention than glycolysis and mitochondrial metabolism, but interest in lipid metabolism in cancer has steadily been increasing over the past few years. Abnormal lipid metabolism is closely associated with tumorigenesis; specifically, abnormal lipid metabolism contributes to invasion and metastasis [131, 132]. The morphological change from an epithelial apical–basal polarized phenotype to a spindle-shaped phenotype, which is closely related to cell membrane fluidity, is the most recognized feature of EMT [30]. Increased plasma membrane fluidity and the destabilization of lipid rafts have been observed during EMT [133] and were ascribed to abnormal lipogenesis and changes in the structural components of lipid rafts modulated by EMT inducers [132, 134].

**Fig. 5:**
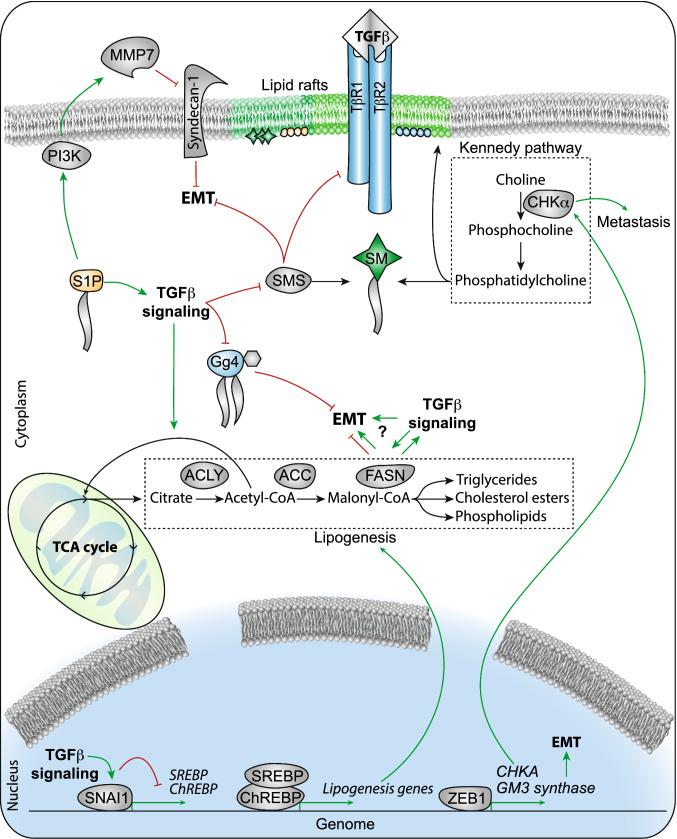
Lipid and choline metabolism in TGFβ-induced EMT. During lipogenesis, citrate from the TCA cycle is transported into the cytoplasm and cleaved by ATP citrate lyase (ACLY) to acetyl-CoA, which is carboxylated to malonyl-CoA by acetyl-CoA carboxylase (ACC). Subsequently, fatty acid synthase (FASN) catalyzes malonyl-CoA into palmitate, a substrate for triglyceride, phospholipid and cholesterol ester synthesis. Sterol regulatory element-binding proteins (SREBP) and carbohydrate-responsive element-binding protein (ChREBP) are important regulators of ACLY, ACC and FASN transcription. Expression of these enzymes and nuclear factors is repressed during TGFβ-induced EMT. Lipid rafts are formed by cholesterol and sphingolipids, including sphingomyelin (SM), sphingosine-1-phosphate (S1P) and glycosphingolipid (GSL). TGFβ-induced EMT correlates with reduced expression of sphingomyelin synthase (SMS), which converts ceramide to SM. This interferes with EMT induction by TGFβ via inhibition of TGFβR1 and SMAD2 phosphorylation. S1P upregulates matrix metalloproteinase 7 (MMP-7) by stimulating the PI3K/AKT signaling pathway. MMP7 is linked to EMT through the regulation of MMP-7/syndecan-1/TGFβ autocrine loop. Furthermore, TGFβ-signaling decreases Gg4, an important component of GSL required for EMT. ZEB1 promotes the expression of a-series glycosphingolipids including GM3 synthase connecting glycosphingolipid metabolism and epithelial cell adhesion during TGFβ-induced EMT. Choline kinase alpha (CHKα) is a key enzyme in the cytidine 5′-diphosphocholine (CDP)-choline pathway (known as the Kennedy pathway) to generate phosphatidylcholine, an essential component of cell membranes. Abnormal expression and activity of CHKα is associated with tumor metastasis. The EMT activator ZEB1-altered choline metabolism by stimulating CHKα

Dysfunctional lipid metabolism in cancer is mainly characterized by enhanced de novo fatty acid synthesis and reduced fatty acid decomposition due to rapid proliferation. Furthermore, enhanced fatty acid metabolism is essential for providing lipids for membrane formation and energy production. For example, expression of fatty acid translocase CD36 elevates intracellular fatty acid levels and promotes EMT of hepatocellular carcinoma (HCC) cells [135]. Fatty acid uptake-related genes, such as caveolin-1 (CAV1) and CD36, are abundantly present in metastatic tumors and have been associated with EMT in multiple cancers [136]. Moreover, CD36-positive cells were essential for metastasis formation in human oral cancer cells [137] and the TGFβ signaling pathway was activated upon free fatty acid addition in hepatocellular carcinoma cells, resulting in EMT induction [135]. Fatty acid synthase (FASN), adenosine triphosphate citrate lyase (ACLY) and acetyl-CoA carboxylase (ACC) are the three main enzymes involved in de novo fatty acid synthesis [138–140]. Increased FASN has been demonstrated in cisplatin-resistant NSCLC cells compared to its expression in parental cells; this difference in expression was deemed responsible for the increased EMT and metastatic potential of cisplatin-resistant cells. FASN suppression impaired EMT with the downregulation of mesenchymal markers via the FASN-TGFβ1-FASN positive loop specifically in cisplatin-resistant cells [141]. However, the opposite observation has also been described. Decreased levels of FASN and ACC were accompanied by the downregulation of carbohydrate-responsive element-binding protein (ChREBP) and sterol regulatory element-binding proteins (SREBP) in NSCLC cells upon TGFβ-induced EMT, two key transcriptional regulators of lipogenesis. Furthermore, SNAI1 suppressed lipogenesis during EMT via inhibiting ChREBP and SREBP and FASN silencing was sufficient to drive EMT and enhance cancer cell metastasis in vivo. In addition, increased oxygen consumption and ATP generation were observed during TGFβ-induced EMT. These effects might be ascribed to the entry of increased acetyl-CoA into the TCA cycle or OXPHOS instead of lipogenesis, generating sufficient ATP for cancer cell migration [142] (Fig. 5).

Except for their de novo fatty acid synthesis, sphingolipids have also been correlated with EMT. Sphingolipids are structural components of the cell membrane with vital roles in cell recognition and signaling [143, 144]. Glycosphingolipids (GSLs) were shown to be involved in TGFβ-induced EMT in NMuMG and HCV29 human normal bladder cells [145]. TGFβ treatment changed the GSL composition and, in particular, downregulated the content of Gg4, which is an important glycosphingolipid that participates in the EMT process. In addition, exogenous supplementation with Gg4 inhibited cell motility and mesenchymal marker expression and elevated the expression of epithelial markers. Furthermore, Gg4 was reported to mediate EMT through its interactions with epithelial molecules such as E-cadherin and β-catenin to maintain epithelial cell membrane organization [146]. The mechanism by which gangliosides regulate EMT may be related to the expression of transcriptional repressors. For instance, ZEB1 upregulates the expression of a-series glycosphingolipids by stimulating the expression of GM3 synthase (St3gal5). Repression of ZEB1 decreased the expression of a-series GSLs and St3gal5, leading to the increased expression of junction-associated proteins such as E-cadherin and plakoglobin [147] (Fig. 5).

Sphingosine-1-phosphate (S1P) is a signaling sphingolipid that is also associated with the EMT process. S1P mediated EMT by regulating the autocrine production of TGFβ1, which contributed to a fibrotic response in A549 lung cancer cells [148]. High levels of serum S1P in hepatocellular carcinoma were found to be associated with poor clinical prognosis [149]. In a recent study, S1P was found to activate PI3K/AKT signaling pathway, resulting in increased MMP-7 expression. MMP-7 regulates the shedding of syndecan-1, a transmembrane heparan sulfate proteoglycan that mediates cell adhesion and migration. The loss of syndecan-1 led to an increase in autocrine TGFβ1, which enabled EMT. Meanwhile, the endogenous expression of TGFβ1 was tightly mediated by MMP-7, thus constituting an MMP-7/syndecan-1/TGFβ1 autocrine loop to mediate hepatocellular carcinoma (HCC) metastasis [150].

Sphingomyelin synthase (SMS), of which there are two isoforms (SMS1/2), are vital enzymes for the production of sphingomyelin (SM) [151, 152]. SM can mediate signaling responses, apoptosis and lipid rafts [153, 154]. The decreased expression of SMS1 was found during TGFβ1-induced EMT in the MDA-MB-231 cell line. In contrast, the overexpression of SMS1-inhibited EMT induced by TGFβ1 and coincides with the upregulation of E-cadherin and downregulation of vimentin [155]. Another study indicated that SMS1 blocks EMT via repressing the expression of TGFβR1 and SMAD2 phosphorylation [155]. Overall, these findings imply that sphingolipids play important roles in EMT induced by TGFβ, and a deeper understanding of the mechanisms by which sphingolipids regulate cancer cell signaling and metastasis will be beneficial to improve cancer treatment.

## Choline metabolism supports EMT

Lipid synthesis is essential for cell growth, proliferation and differentiation, and although many mutations in oncogenes, such as KRAS and MYC, control lipid metabolism [156, 157], few lipid enzymes have been reported to drive tumorigenesis [158]. Choline kinase α (CHKα), a key enzyme involved in choline metabolism, is a notable exception [159]. Aberrant choline metabolism has been observed in multiple cancers. An increasing amount of research has focused on the molecular mechanism underlying abnormal choline metabolism, providing potential targets in cancer therapy [158, 160].

CHKα belongs to the choline kinase protein family, a group of enzymes with a choline kinase/ethanolamine kinase active domain encoded by the *CHKA* and *CHKB* genes [159]. CHKα, the first enzyme in the cytidine 5′-diphosphocholine (CDP)-choline pathway (also known as the Kennedy pathway), catalyzes the phosphorylation of free choline using ATP as a phosphate donor to generate phosphocholine. Phosphocholine is a substrate in the synthesis of phosphatidylcholine, the major phospholipid in cell membranes. Numerous studies have confirmed that the abnormal expression and activity of CHKα are apparent in many cancers and correlated with metastasis [161–163].

Although direct regulation of CHKα by TGFβ-signaling has not been reported to date, the expression of EMT transcription factors, including ZEB1, TWIST1 and SNAI1, and the EMT target genes vimentin and N-cadherin were found to be associated with dysfunctional choline metabolism in glioblastoma cells. Also, inhibition of CHKα significantly reduced cell viability, invasiveness, clonogenicity, and the expression of EMT-associated genes [164]. In ovarian cancer cells, downregulation of CHKα impaired aggressiveness and enhanced cellular sensitivity to drug treatment [165]. Furthermore, a combination of *CHKA* loss and 5-fluorouracil (5-FU) treatment resulted in a larger reduction of cell viability and proliferation in breast cancer cell lines compared to that following 5-FU treatment alone [166]. Overexpression of CHKα induced an aggressive phenotype and increased resistance to 5-FU treatment in MCF-7 breast cancer cells [167]. In addition, increased levels of CHKα significantly reduced the survival of mice with bladder cancer, while the inhibition of CHKα suppressed tumor growth and resulted in a relevant increase in in vivo survival [168]. Further research found that the EGFR/PI3K/AKT pathway was essential for mediating CHKα function in colorectal carcinoma [169]. Inhibition of CHKα by EB-3D, a selective CHKα inhibitor, induced senescence in breast cancer cell lines through stimulation of the 5′ AMP-activated protein kinase (AMPK)-mTOR pathway and impaired breast cancer cell proliferation, migration, and invasion accompanied by the loss of mesenchymal markers [170].

Due to the increased expression and activity of CHKα in many different cancers described above, CHKα has become a promising target in cancer therapy. Among many CHKα inhibitors under development, RSM-932A is the first inhibitor to be tested in humans in a phase I clinical trial conducted by TCD Pharma (Valladolid, Spain) at two U.S. clinical centers (NCT01215864) in patients with advanced solid tumors.

## Conclusion and perspectives

EMT is an important stage of cancer development. Cancer cells undergoing EMT acquire invasive characteristics and infiltrate into the surrounding matrix to form a microenvironment that promotes tumor growth and metastasis. As summarized above, increasing evidence supports that EMT is accompanied by complex metabolic rewiring. Changes in metabolites and pathways might be used to improve cancer diagnosis, and the targeting of cancer metabolism to prevent EMT may hold promising new therapeutic strategies for cancer patients.

Some molecules have successfully been applied in clinical therapy to target metabolic pathways. For instance, a group of antisense nucleoside analogs, 5-fluorouracil, gemcitabine, methotrexate and fludarabine, which target different enzymes involved in nuclei acid synthesis, acted as an effective therapy for diverse cancers in patients [171]. Another example is l-asparaginase, an agent approved to treat acute lymphoblastic leukemia (ALL) and non-Hodgkin’s lymphoma via catalyzing the conversion of l-asparagine to l-aspartic acid and ammonia, limiting the asparagine available for the tumor [64]. In addition to the successful use of molecules targeting metabolism in the clinic, several metabolic enzymes are evolving as potential candidate targets. Examples include but are not limited to proteins closely linked to TGFβ-induced EMT, such as GLUT1, HK2, PFKFB3, PKM2, LDHA and PDK4, proteins that control glycolysis [172–180], and FASN and ACC, which are involved in lipid synthesis [181–184] (Table 1). Overall, a large number of preclinical research reports provide alternative therapeutic strategies that target EMT and tumor cell migration.

**Table 1:** Potential metabolic target of cancer therapy

Target	Description	Agent	Development stage	Indication	References
GLUT1	Glycolysis: glucose transportation	WZB117, BAY-876	Preclinical data	Anticancer activity in vitro and in vivo	[169, 170]
HK2	Glycolysis: phosphorylation of glucose to glucose-6-phosphate (G6P)	2-Deoxyglucose, lonidamine	Preclinical and clinical data	Anticancer activity in vitro and in vivo; Lonidamine has been tested in phase 3 clinical trial (NCT00435448)^a^	[171, 172]
PFKFB3	Glycolysis: activator of PFK1, promote glycolysis	ACT-PFK158	Preclinical and clinical data	Anticancer activity in vitro and in vivo, has been tested in phase 1 clinical trial (NCT02044861)	[173]
PKM2	Glycolysis: dephosphorylation of phosphoenolpyruvate to pyruvate	TLN-232	Preclinical and clinical data	Anticancer activity in vitro and in vivo, has been tested in phase 2 clinical trial (NCT00735332)	[174, 175]
LDHA	Glycolysis: conversion of pyruvate to lactate	FX11, GNE-140	Preclinical data	Anticancer activity in vitro and in vivo	[169, 176]
PDK4	Glycolysis: a negative regulator of PDH	Dichloroacetate	Preclinical and clinical data	Anticancer activity in vitro and in vivo, has been tested in phase 2 clinical trial (NCT01386632)	[177]
FASN	Lipogenesis: synthesis of palmitate from malonyl-CoA	TVB–2640	Preclinical and clinical data	Anticancer activity in vitro and in vivo, has been tested in phase 2 clinical trial (NCT03179904)	[178–180]
ACC	Lipogenesis: carboxylation of acetyl-CoA to malonyl-CoA	GS-0976	Preclinical and clinical data	Decreased hepatic steatosis, has been tested in phase 2 clinical trial (NCT02856555)	[178, 179, 181]

^a^Clinical trial information was obtained from https://clinicaltrials.gov/

Targeting TGFβ directly during EMT and accompanying metabolic rewiring might provide a potential window for cancer treatment as recent studies have shown that TGFβ inhibition reverses therapy resistance, enhances combinatorial synergy and sensitizes for radiotherapy in cell and mouse models [18]. Systemic treatment with TGFβ inhibitors, however, comes with side effects due to the pleiotropic nature of the pathway and, therefore, is unsuitable or challenging for use in the clinic [18]. In turn, there is a need to find the exact tumor supporting downstream effects of TGFβ signaling to provide promising opportunities for targeting and with this review, we put metabolic reprogramming in the spotlight. However, targeting metabolism comes with a new set of challenges.

Rather than a single metabolite, multiple metabolic pathways are changed simultaneously in tumorigenesis, and combined these metabolic pathways provide potent support for tumor progression. Tumor cells possess high metabolic plasticity in their use of metabolic pathways which also depend on a dynamically changing tumor microenvironment. Normal tissues also depend on the same metabolic pathways for survival and proliferation. These characteristics represent a big challenge for the therapeutic targeting of tumor metabolism. Increased glycolysis as part of the Warburg effect is common in cancer cells but no inhibitors of glycolysis have been approved for therapy, as these also target glycolysis in all healthy cells [171]. In addition, glutamine metabolism can compensate for the decreased glycolysis in tumor cells. Thus, partial and/or combinational targeting might be a more effective strategy as it can be expected that only the highly dependent tumor cells will be affected. Alternatively, instead of focusing on blocking main metabolic routes, targeting synthesis of tumor-specific metabolites that are rare in healthy cells could be more promising. Metabolic changes during EMT are interesting in this respect as EMT is an almost unique property of cancer cells in the adult body, with small cell populations during wound healing and angiogenesis as exceptions.

Finally, next to metabolic changes in cancer cells during TGFβ-induced EMT, the metabolic microenvironment will be affected. This not only contributes to promote tumor progression and therapy resistance, but also connects to immune suppression. Nutrient deficiency caused by hyperactive tumor cell metabolism and metabolites released from tumor cells into the microenvironment affect immune cell function and facilitate immune suppression [185, 186]. Increased glycolysis in tumor cells results in low glucose levels in the microenvironment and is linked to inhibition of glycolysis in tumor-infiltrating lymphocytes [187]. Additionally, accumulation of lactate in tumor microenvironment impairs proliferation and mobility of cytotoxic T lymphocytes and natural killer (NK) cells [56]. Conclusion, this review illustrates that the understanding and targeting of TGFβ-induced metabolic changes during EMT is emerging and provides basis for novel cancer treatments including targeted and immune therapy.

## Abbreviations

2HG: R-2-Hydroxyglutarate
5-FU: 5-Fluorouracil
ACC: Acetyl-CoA carboxylase
ACLY: Adenosine triphosphate citrate lyase
ALL: Acute lymphoblastic leukemia
AMPK: 5′ AMP-activated protein kinase
AT-RvD1: Aspirin-triggered resolvin D1
CAV1: Caveolin-1
CDKs: Cyclin-dependent kinases
CDP: Cytidine 5′-difosfocholine
CHKα: Choline kinase
ChREBP: Carbohydrate-responsive element-binding protein
CRC: Colorectal cancer
E-cadherin: Epithelial cadherin
DPYD: Dihydropyridine dehydrogenase
EGFR: Epidermal growth factor receptor
EMT: Epithelial-to-mesenchymal transition
ERK: Extracellular signal-regulated kinase
F1,6P2: Fructose-1,6-bisphosphate
F2,6P2: Fructose-2,6-bisphosphate
F6P: Fructose-6-phosphate
FASN: Fatty acid synthase
FAD: Flavin adenine dinucleotide
FH: Fumarate hydratase
G6P: Glucose-6-phosphate
GLUT1: Glucose transporter 1
GLUT3: Glucose transporter 3
GSLs: Glycosphingolipids
GST: Glutathione S-transferase
HCC: Hepatocellular carcinoma
HMGA2: High-mobility group AT-hook 2
HIF: Hypoxia-inducible factor
HK2: Hexokinase 2
IDH: Isocitrate dehydrogenase
KG: Ketoglutarate
LDHA: Lactate dehydrogenase type A
LSH: Lymphoid-specific helicase
MAPK: Mitogen-activating protein kinase
MEK: MAPK kinase
MMP: Matrix metalloproteinase
mTOR: Mammalian target of rapamycin
NADH: Nicotinamide adenine dinucleotide
NF-κB: Nuclear factor-κB
NK: Natural killer cells
NMuMG: Namru murine mammary gland
NOX4: NADPH oxidase 4
NPC: Nasopharyngeal carcinoma
NQO1: Quinone oxidoreductase 1
NRF2: Nuclear factor E2-related factor 2
NSCLC: Non-small cell lung carcinoma
OXPHOS: Oxidative phosphorylation
PDAC: Pancreatic ductal adenocarcinoma
PDH: Pyruvate dehydrogenase
PDK: Pyruvate dehydrogenase kinase
PEP: Phosphoenolpyruvate
PFK1: 6-Phosphofructo-1-kinase
PFKFB3: 6-Phosphofructo-2-kinase/fructose-2,6-biphosphatase 3
PI3K: Phosphatidyl inosititol 3-kinase
PKM2: Pyruvate kinase M2
PTEN: Phosphatase and tensin homolog
PTP1B: Protein–tyrosine phosphatase 1B
ROS: Reactive oxygen species
SDH: Succinate dehydrogenase
SMAD: Sma and Mad related
SMS: Sphingomyelin synthase
SOD: Superoxide dismutase
S1P: Sphingosine-1-phosphate
SREBP: Sterol regulatory element-binding protein
St3gal5: GM3 synthase
TCA: Citric acid cycle
TET: Ten–eleven translocation
TGFβ: Transforming growth factor β
TGFβR1: TGF-β type I receptor
TGFβR2: TGF-β type II receptor
TGIF2: TGFβ-induced factor homeobox 2
TXN2: Mitochondrial thioredoxin
VEGF: Vascular endothelial growth factor
